# Zeolitic Imidazolate Framework-8 Composite-Based Enzyme-Linked Aptamer Assay for the Sensitive Detection of Deoxynivalenol

**DOI:** 10.3390/bios13090847

**Published:** 2023-08-25

**Authors:** Zaixi Shu, Run Zhou, Guijie Hao, Xingyue Tang, Xin Liu, Jie Bi, Huang Dai, Yafang Shen

**Affiliations:** 1 College of Food Science and Engineering, Wuhan Polytechnic University, Wuhan 430023, China; shuzaixi@163.com (Z.S.); zhourun285@163.com (R.Z.); 15327248225@163.com (X.T.); liuxinhook@126.com (X.L.); bj15926225191@163.com (J.B.); 2 Key Laboratory for Deep Processing of Major Grain and Oil, Wuhan Polytechnic University, Ministry of Education, Wuhan 430023, China; 3 Key Laboratory of Healthy Freshwater Aquaculture, Ministry of Agriculture and Rural Affairs, Key Laboratory of Fish Health and Nutrition of Zhejiang Province, Huzhou Key Laboratory of Aquatic Product Quality Improvement and Processing Technology, Zhejiang Institute of Freshwater Fisheries, Huzhou 313001, China; haoguijie2022@hotmail.com

**Keywords:** deoxynivalenol, zeolitic imidazolate framework-8, biomimetic mineralization, enzyme-linked aptamer assay, colorimetric detection

## Abstract

The mycotoxin deoxynivalenol (DON) is a prevalent contaminant in cereals that threatens the health of both humans and animals and causes economic losses due to crop contamination. The rapid and sensitive detection of DON is essential for food safety. Herein, a colorimetric biosensor based on horseradish peroxidase- and gold nanoparticle-encapsulated zeolitic imidazolate framework-8 (HRP&Au@ZIF-8) was developed for the sensitive screening of DON. The synthesized HRP&Au@ZIF-8 probes not only held great potential for signal amplification but also exhibited stable catalytic activity even under extreme conditions, which endowed the biosensor with both good sensitivity and stability. Under the optimized conditions, qualitative measurement of DON can be achieved through visual inspection, and quantitative evaluation can be performed via absorbance measurements at a characteristic wavelength of 450 nm. The proposed method has demonstrated high sensitivity with a linear detection range of 1–200 ng/mL and a detection limit of 0.5068 ng/mL. It also presented good selectivity and reliability. Furthermore, DON in spiked cereal samples has been quantified successfully using this method. This novel approach demonstrates significant potential for the facile and expeditious detection of DON in cereal products and brings us one step closer to enhancing food safety.

## 1. Introduction

Mycotoxins are a group of poisonous secondary metabolites produced by fungi (e.g., *Fusarium*, *Penicillium* and *Aspergillus*) and can cause physiologically toxic effects on animal and human health [1]. Among various mycotoxins, deoxynivalenol (DON) is a ubiquitous contaminant found in cereal grains. It is naturally produced by several *Fusarium* spp. such as *Fusarium oxysporum* and *Fusarium graminearum* during their growth in cereals under certain temperatures and humidity [2]. DON can enter the food supply chain through contaminated food and feed, bringing carcinogenic, teratogenic and hepatotoxic hazards to both animals and humans [3,4]. Therefore, detecting DON accurately in food is vital to maintaining food safety and ensuring public health.

In order to detect DON, numerous instrumental methods, as represented by high-performance liquid chromatography (HPLC) [5] and thin-layer chromatography [6] have been well established. These methods are accurate and reliable but also exhibit many limitations, for instance, complicated sample preparation and laborious and time-consuming operation, as well as the requirement of well-skilled operators and sophisticated equipment. These shortcomings make them incapable of rapidly detecting samples in large numbers and fail to meet the requirement of household daily testing, grassroots supervision and market testing. As an alternative, the enzyme-linked colorimetric methods, with the merits of simplicity, rapidity and naked eye-recognizable signal output, have a higher possibility of in-field application [7,8]. Therefore, they have garnered widespread attention and usage in the realm of food safety [9,10].

In colorimetric methods, horseradish peroxidase (HRP) is among the most frequently used enzymes owing to their overwhelming advantages of extremely high catalytic activity and easy labeling. However, the catalytic activity of HRP is easily influenced by storage and operational conditions, which affects the performance of colorimetric analysis [11]. Extreme conditions including thermal stress, excessively acidic or alkaline environment and organic solvents significantly decrease the catalytic activity of HRP molecules. And the decreased enzymatic activity always hampers the sensitivity and reliability of colorimetric methods in practical applications. To address this problem, an enormous amount of effort has been devoted to developing HRP-based composites for the improvement of HRP’s stability [12]. A wide variety of nanomaterials including metal–organic frameworks (MOFs) [13], multi-walled carbon nanotubes [14] and graphene oxide [15], with ease of preparation, excellent thermal stability as well as great versatility, have been used for enzyme immobilization. Particularly, a great deal of attention has been drawn to MOFs for their remarkable properties of ultra-high porosity, good biocompatibility, multi-metal sites and large surface area [16,17,18,19]. Among the huge family of MOFs, zeolitic imidazolate framework-8 (ZIF-8), synthesized by the coordination between zinc ions and 2-methylimidazole (2-MI), is considered to be a feasible carrier for the encapsulation/immobilization of enzymes because of its open structure, high porosity and satisfactory thermostability [20,21]. It has been widely used as a protective coating for biomolecules, maintaining the biological activities of biomolecules in harsh environments [22]. Therefore, the implementation of MOF–enzyme composites can possibly overcome the fragility of HRP in colorimetric assays.

Aptamers (Apt) are a kind of functional nucleic acid that show high specificity and affinity towards their targets. They offer various advantages over antibodies such as high stability, non-immunogenicity, cost-effectiveness in preparation as well as suitability for use in harsh conditions [23]. Apt have the ability to fold into distinct secondary or tertiary structures when exposed to specific environmental conditions and specifically bind to various types of compounds from small molecules to entire cells [24]. Therefore, Apt are good alternatives to antibodies as recognition elements and have been extensively used in different fields of environmental monitoring, biological analysis, food safety, etc. [25,26]. With the merits of the ZIF-8’s big specific area, the high density of the recognition molecules conjugated on the ZIF-8 surface will accelerate the binding reaction. Meanwhile, the coating of ZIF-8 on the HRP surface can enhance the stability and sensitivity of colorimetric detection. These inspired us to develop an enzyme-linked aptamer assay (ELAA) based on the HRP@ZIF-8 composites to enhance the sensitivity and reliability of DON detection.

In this work, gold nanoparticles (AuNPs)-embedded HRP@ZIF-8 (HRP&Au@ZIF-8) composites were synthesized as signal transducers to construct an ELAA for the detection of DON. DNA Apt for DON were used as the recognition element. Signal tags were prepared by labeling the complementary DNA (cDNA) Apt on the HRP&Au@ZIF-8 surface. In this signal tag, HRP served as a reporter to produce colorimetric signals. Utilizing the merits of high surface area and pore encapsulation, ZIF-8 was used as an effective carrier for HRP, protecting the enzyme against harsh conditions. Additionally, the introduced AuNPs offered abundant binding sites for cDNA, and the immobilized cDNA acted as competitors to the aptamers’ binding sites with target DON. The HRP&Au@ZIF-8-based ELAA was successfully used for the determination of DON in spiked cereal samples, and satisfactory results were obtained. This method innovatively utilized the HRP&Au@ZIF-8/cDNA composites and aptamers as signal probes and recognition elements, respectively, to overcome the disadvantage of the instability of conventional enzyme-linked immunosorbent assay (ELISA). It enables DON detection even under harsh environments, for example, the detection of DON during transportation or in remote resource-limited regions where temperature and other environmental factors are uncontrollable. To the best of our knowledge, this is the first report that utilized the HRP&Au@ZIF-8/cDNA nanocomposites for the detection of DON.

## 2. Materials and Methods

### 2.1. Reagents

Trisodium citrate, chloroauric acid (HAuCl_4_) and Tween-20 were purchased from Sinopharm Chemical Reagent Co., Ltd. (Shanghai, China). Streptavidin, Zn(NO_3_)_2_·6H_2_O, 2-MI, 3,3′,5,5′-Tetramethylbenzidine (TMB) and HRP, high binding 96-well microplates (white) were purchased from Wuhan Fei Yang Biotechnology Co., Ltd. (Wuhan, China). Dipotassium hydrogen phosphate, potassium dihydrogen phosphate, potassium sulfate and other chemicals of analytical grade were bought from Tianjin Chemical Reagent Company (Tianjin, China). DON, nivalenol (NIV), 3-acetyl-deoxynivalenol (3-ACDON), 15-acetyl-deoxynivalenol (15-ACDON), aflatoxin (AFB_1_, AFB_2_, AFG_1_, AFG_2_) and ochratoxin A (OTA) were obtained from Shandong Lvdu Biotechnology Co., Ltd. (Binzhou, China). Apt, cDNA and other oligonucleotides were all synthesized by Sangon Biotech Co., Ltd. (Shanghai, China), and their sequence and modification information is provided in Table S1. Deionized water treated by a Milli-Q system (18.2 MΩ cm, Millipore, Billerica, MA, USA) was used throughout the tests. All of the other reagents were analytical or better grade and directly used without further purification. Unless otherwise noted, all tests were conducted at room temperature.

### 2.2. Apparatus

The absorbance spectra were collected using a multi-functional microplate reader (PerkinElmer, Singapore). Scanning electron microscopy (SEM, Zeiss Gemini 300, Oberkochen, Baden-Württemberg, Germany) was utilized for the characterization of nanomaterials’ microstructure and surface morphologies. Energy dispersive spectrometry (EDS) analysis and elemental mapping were performed by SEM with EDS capability. Fourier transform infrared spectroscopy (FTIR, Nicolet IS10, Thermo Fisher Scientific Co., Ltd., Waltham, MA, USA) and Raman spectroscopy (inVia Qontor; Renishaw Co., Ltd., Gloucestershire, London, UK) were implemented in order to obtain the chemical bond information. The crystalline phase and thermal stability were characterized via an X-ray diffractometer (XRD, Empyrean, Billerica, Massachusetts, German) and a thermogravimetric analyzer (TGA4000, Mettler-Toledo Co., Ltd., Zurich, Switzerland), respectively.

### 2.3. Preparation of the AuNPs and HRP&Au@ZIF-8/cDNA Composites

AuNPs were synthesized according to a classical citrate reduction method [7]. In brief, 40 mL of 1% aqueous sodium citrate solution (*w*/*w*) was rapidly injected into 1000 mL of the boiling aqueous HAuCl_4_ solution (0.1%, *w*/*v*) under vigorous stirring until a wine-red colloidal gold was formed. According to the SEM image, the synthesized AuNPs show an average diameter of about 18 nm (Figure S1). In order to prepare the HRP&Au@ZIF-8/cDNA composites, 0.0892 g of Zn(NO_3_)_2_·6H_2_O and 5 mg of HRP were dissolved in 5 mL of deionized water and then mixed with 5 mL of the as-prepared AuNPs solution. The mixture was then slowly added to 10 mL of 2 M 2-MI, magnetically stirred for 1 h and left to stand overnight. The crude HRP&Au@ZIF-8 products were centrifuged at 11,700 g (3H24RI, Heraeus, Hanau, Germany) for 15 min, collected, washed with deionized water and redispersed in 6 mL of 10 mM phosphate-buffered saline (PBS, pH 7.4). Then, 600 μL of 500 nM cDNA was added to the HRP&Au@ZIF-8 dispersion liquid and incubated at 37 °C for 4 h with continuous shaking. After removing the unbound cDNA by centrifugation and triple washing with 10 mM PBS, the obtained HRP&Au@ZIF-8/cDNA composites were re-dispersed in 6 mL of 10 mM PBS and stored in a 4 °C refrigerator for further use.

### 2.4. Construction of the Colorimetric ELAA Based on HRP&Au@ZIF-8/cDNA for DON Detection

The 96-well plate wells were coated with 100 μL of streptavidin solution and incubated at 37 °C for 1 h. After being emptied and washed thrice with PBS containing 0.05% Tween-20 (PBST, pH 7.4), each well was sealed with a mixture of 1.5% BSA-PBST and 100 nM single-stranded DNA (Table S1) at 37 °C for 1 h to reduce nonspecific adsorption. After discarding the supernatant and washing for three times, 100 μL of 50 nM biotin-Apt solution was transferred to the wells for Apt coating. The plate wells were incubated at 37 °C for 1 h and washed again to remove the unbound biotin-Apt. For detection, the DON samples were added to the well and reacted at 37 °C for 1 h. Then, the unreacted DON was cleared away after three times of washing. HRP&Au@ZIF-8/cDNA solution was added to the well and bound to the residual Apt. After a one-hour incubation at 37 °C, the unreacted HRP&Au@ZIF-8/cDNA probes were removed thoroughly by washing the wells with PBST three times. After that, 50 μL of ready-to-use TMB solution was added and the reaction was stopped after the addition of 50 μL of 2 M H_2_SO_4_. After 5 min, the color of the solution changed, and the DON was qualitatively detected via observation of the naked eye color change. Quantitative detection was also achieved by absorbance measurements at 450 nm via a multi-functional microplate reader. All measurements were carried out at least three times.

### 2.5. Preparation of the Spiked Cereal Samples

Three cereal products including rice, wheat and maize were all purchased from a local supermarket (Wuhan, China). Sample extraction was conducted referring to our recent work [7]. The extracts were diluted five times with 10 mM PBS (pH 7.4) and spiked with DON at different concentration levels. Then, DON concentrations in the spiked cereal samples were determined using the same procedure as described above. In addition, a commercial DON ELISA kit was utilized to detect the concentrations of DON in the spiked samples in parallel to evaluate the differences between the established method and the commercial kit.

### 2.6. Statistical Analysis

All the data were processed and analyzed using SPSS 25.0 software (SPSS Incorporated, Chicago, IL, USA). A one-way ANOVA was performed for statistical significance analysis with a significance threshold of *p* < 0.05.

## 3. Results and Discussion

### 3.1. Detection Principle of the Colorimetric ELAA Based on HRP&Au@ZIF-8/cDNA

**Scheme 1:**
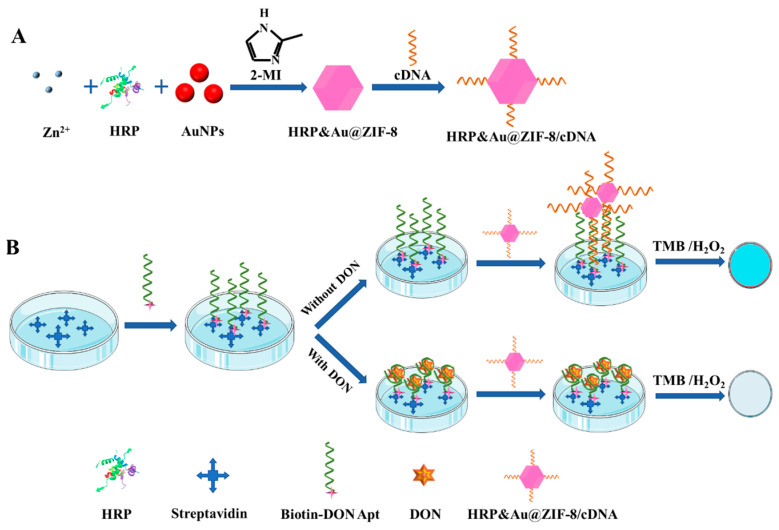
Schematic diagram of DON detection by the proposed ELAA based on HRP&Au@ZIF-8. The preparation process of the HRP&Au@ZIF-8/cDNA probe (**A**) and the detection principle (**B**).

HRP is among the most commonly used enzymes for signal transduction, which can effectively catalyze the colorless substrate TMB into soluble blue substrates (TMBox) in the presence of H_2_O_2_. As shown in Scheme 1A, to enhance the stability of the enzyme, HRP and AuNPs were co-encapsulated in the cavity of ZIF-8 via a biomimetic mineralization method, resulting in the formation of HRP&Au@ZIF-8 nanocomposites. Then, sulfhydrylated cDNA was attached to the surface of HRP&Au@ZIF-8 via Au–S bond to prepare HRP&Au@ZIF-8/cDNA probes. The co-encapsulation of HRP and AuNPs in ZIF-8 improves the stability of HRP in extreme environments and also provides multiple binding sites for cDNA immobilization. Meanwhile, the biotin-modified DON Apt was immobilized on the microplates through its strong affinity towards the precoated streptavidin. DON in samples was first bound to DON Apt. After the HRP&Au@ZIF-8/cDNA signal tag was added to the wells, the unbound DON Apt formed a duplex strand with its cDNA and captured the signal tags on the well surface. Then, the ready-to-use TMB solution was poured into each of the wells for HRP catalysis and colorimetric signal output. Finally, the color change of the solution can be observed with the naked eye, and the absorbance values at 450 nm were measured after adding H_2_SO_4_ for the quantitative detection of DON (Scheme 1B). Under the condition that a fixed number of Apt was immobilized on the microplate well, the amount of HRP&Au@ZIF-8/cDNA probes captured by the Apt was negatively related to the content of the DON. Therefore, the absorbance values derived from HRP catalysis decreased with the increased concentration of DON.

### 3.2. Characterization of the HRP&Au@ZIF-8 Composites

**Figure 1:**
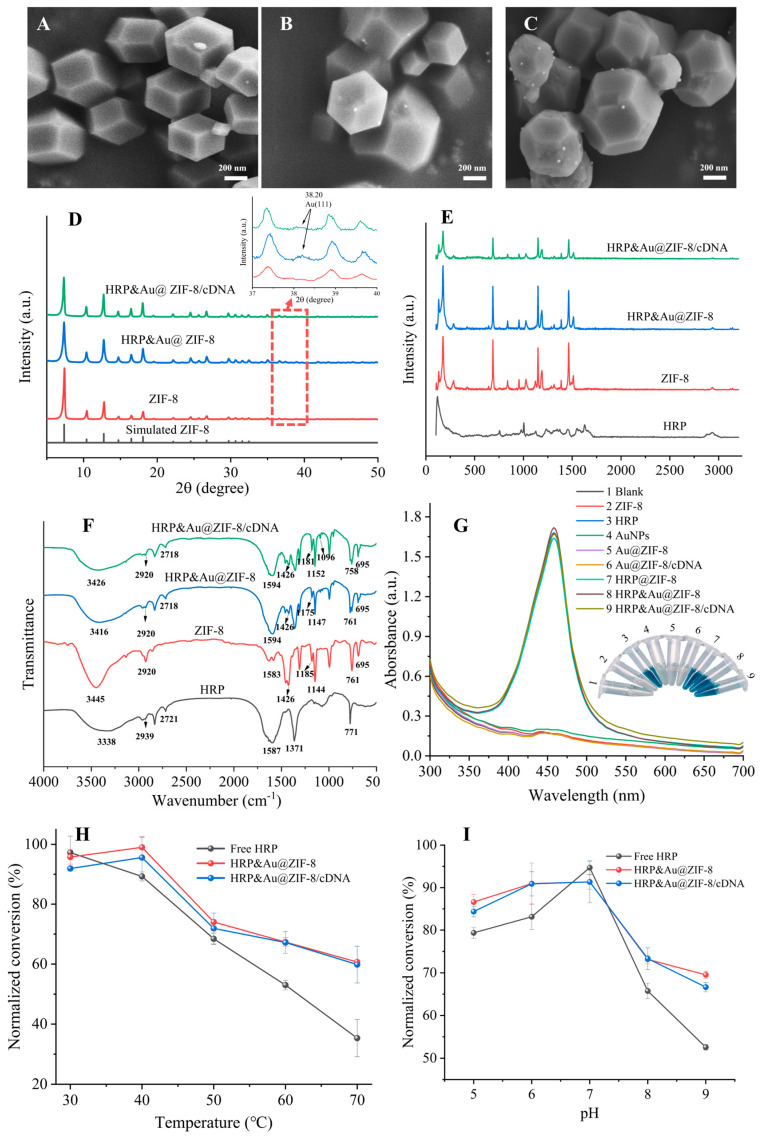
SEM images of ZIF-8 (**A**), HRP&Au@ZIF-8 (**B**) and HRP&Au@ZIF-8/cDNA (**C**); XRD (**D**), Raman spectra (**E**), FTIR spectra (**F**) and UV-vis characterization (**G**) of the signal tag; the effect of temperature (**H**) and pH (**I**) on the catalytic performance of HRP&Au@ZIF-8/cDNA. Product conversion was normalized against the activity of free HRP molecules at room temperature.

As the SEM images show in Figure 1A–C, the diameters of pristine ZIF-8, HRP&Au@ZIF-8 and HRP&Au@ZIF-8/cDNA were (445.11 ± 64) nm, (472.22 ± 40) nm and (485.70 ± 70) nm, respectively. ZIF-8 showed a smooth uniform rhombic dodecahedron geometry with no significant changes in its morphology before and after the encapsulation of HRP and AuNPs. In Figure 1B,C, it was obvious that AuNPs were successfully modified on ZIF-8. In comparison with ZIF-8, the morphology of HRP&Au@ZIF-8 and HRP&Au@ZIF-8/cDNA did not change significantly, indicating that the modification of HRP, AuNPs and cDNA would not damage the structure of ZIF-8. The EDS mapping images in Figure S2 indicated that the elements of C (58.79%), N (18.04%), O (2.64%), Zn (20.36%) and Au (0.17%) were uniformly distributed in the HRP&Au@ZIF-8 nanocomposites. XRD diffraction was used to verify the crystallinity of nanocomposites. As shown in Figure 1D, the XRD spectrum of ZIF-8 displayed six characteristic peaks located at 7.21°, 10.43°, 12.78°, 14.82°, 16.48° and 18.15°, which was consistent with the (110), (200), (211), (220), (310) and (222) planes of the simulated ZIF-8, confirming the highly crystalline structure of ZIF-8 [20]. The presence of AuNPs yielded a standard peak (38.20°) associated with Au (111) [27,28], as shown in the inset of Figure 1D. Meanwhile, the peaks of HRP&Au@ZIF-8 and HRP&Au@ZIF-8/cDNA were highly similar to the peaks in ZIF-8, indicating that HRP and AuNPs did not destroy the crystal structure of ZIF-8 [29]. Raman spectroscopy was also used for the characterization of chemical bonds. Figure 1E shows the Raman spectra of HRP, ZIF-8, HRP&Au@ZIF-8 and HRP&Au@ZIF-8/cDNA nanocomposites. The band at 1500 cm^−1^ was associated with C=C stretching vibration, while those in the 1550–1580 cm^−1^ region were mainly from N=N stretching vibration [18]. Additionally, the Raman bands ranging from 2800 to 3000 cm^−1^ resulted from C-H stretching vibrations. Furthermore, strong Raman bands from imidazole and Zn-N could be observed in the 687–1142 cm^−1^ range [30], indicating the successful synthesis of ZIF-8. The Raman spectra of the HRP&Au@ZIF-8 and HRP&Au@ZIF-8/cDNA composites retained the characteristic bands of ZIF-8, implying that HRP and AuNPs encapsulation, as well as cDNA conjugation, would not cause structure damage to ZIF-8. It should be noted that the characteristic Raman peaks of HRP and cDNA, such as the amide bands of HRP, were not observed in the Raman spectra of HRP&Au@ZIF-8/cDNA. We speculated that these Raman peaks were too weak to be observed as a result of their low content levels. Figure 1F shows the FTIR spectra of HRP, ZIF-8, HRP&Au@ZIF-8 and HRP&Au@ZIF-8/cDNA, respectively. The pure ZIF-8 showed peaks at several wavenumbers such as 3445 cm^−1^, 1426 cm^−1^, 2920 cm^−1^, 761 cm^−1^ and 695 cm^−1^, corresponding to N-H, C-N, C-H, Zn-O and Zn-N vibrations, which was in accordance with the reported FTIR data of ZIF-8 [18,31,32,33]. The FTIR spectra of HRP&Au@ZIF-8 and HRP&Au@ZIF-8/cDNA exhibited similar characteristic peaks with those of ZIF-8, further evidencing that the encapsulation of AuNPs and HRP did not affect the chemical structure of ZIF-8. In addition, the peak located at 1594 cm^−1^ for HRP&Au@ZIF-8 and HRP&Au@ZIF-8/cDNA belonged to the amide II band in HRP, implying the successful immobilization of HRP in the nanocomposites [34]. Finally, UV-vis characterization was conducted in order to verify the presence of cDNA on HRP&Au@ZIF-8/cDNA. As shown in Figure S3, the UV-vis spectrum of HRP&Au@ZIF-8/cDNA owned a characteristic absorption peak of cDNA at 260 nm, indicating that cDNA was successfully modified on the surface of HRP&Au@ZIF-8.

To verify the thermal stability of the nanocomposites, thermogravimetric analysis (TGA) was performed under nitrogen flow (Figure S4). ZIF-8 lost 14% of its original mass from 28 °C to 166 °C as a result of the removal of guest molecules (mainly H_2_O) and the unreacted 2-MI from the surface cavities of the nanocrystals [35]. Thereafter, the TGA trace of ZIF-8 displayed a long plateau from 166 °C to 430 °C, indicating that ZIF-8 has good thermal stability. As for HRP@ZIF-8 and HRP&Au@ZIF-8, the TGA curves of these two nanocomposites reached a plateau without considerable mass loss in the temperature range of 200–500 °C, demonstrating their good thermal stability under this high temperature range. Then, they showed a gradual decomposition from 500 °C to 600 °C, resulting in a weight loss of approximately 8.7%, which was attributed to the degradation of the encapsulated HRP enzymes [17]. Thereafter, the nanocomposites began to disintegrate and induced a great mass loss. The TGA curves also showed that HRP@ZIF-8 and HRP&Au@ZIF-8 had a higher decomposition temperature than that of pure ZIF-8, indicating that the encapsulation of HRP may further improve the thermal stability of ZIF-8.

Then, UV-vis characterization was performed to investigate the catalytic ability of the nanocomposites. The UV-vis absorption spectra showed that HRP, HRP@ZIF-8, HRP&Au@ZIF-8 and HRP&Au@ZIF-8/cDNA were all able to catalyze TMB oxidation, turning the colorless solution into a blue one, but ZIF-8, AuNPs, Au@ZIF-8 and Au@ZIF-8/cDNA did not show obvious catalytic activity in this reaction system. After adding the termination fluid, the solution containing HRP, HRP@ZIF-8, HRP&Au@ZIF-8 and HRP&Au@ZIF-8/cDNA displayed a distinctive characteristic peak at 450 nm (Figure 1G). This implied that the porous structure of ZIF-8 enabled an efficient mass transfer, which allowed the chromogenic substrate TMB/H_2_O_2_ to react in close proximity to the encapsulated HRP. Meanwhile, the similar absorbance peak generated by HRP&Au@ZIF-8/cDNA catalysis indicated that the modification of cDNA did not affect the catalytic ability of HRP&Au@ZIF-8. The above results indicated that the HRP&Au@ZIF-8/cDNA composites could maintain the catalytic activity of HRP.

The enzymatic kinetic properties of the HRP&Au@ZIF-8/cDNA composites were analyzed according to the Michaelis–Menten model [36]. The apparent Michaelis–Menten constant (*K*_m_) and the maximum reaction velocity (*V*_max_) were determined to be 1.36 mM and 8.39 × 10^–2^ mM/s, respectively, from the Lineweaver–Burk plot (Figure S5). To estimate the protective performance of ZIF-8 in HRP&Au@ZIF-8 and HRP&Au@ZIF-8/cDNA composites, the effects of temperature and pH on the catalytic activity of the composites were evaluated. As shown in Figure 1H, HRP&Au@ZIF-8 and HRP&Au@ZIF-8/cDNA composites maintained the activity of HRP in the temperature range of 30–70 °C, and both composites displayed an approximately 67% conversion of TMB to TMBox at 70 °C. Meanwhile, only 35.34% substrate conversion for free HRP was achieved under the same temperature condition. The enhanced tolerance to a wider temperature range of HRP&Au@ZIF-8 and HRP&Au@ZIF-8/cDNA composites was most likely attributed to ZIF-8’s rigid structure in which HRP molecules were encapsulated tightly and able to maintain their structure even in high temperatures [37]. As shown in Figure 1I, the optimum pH for free HRP was 7. When adjusting the pH values to 5, 6, 8 and 9, the free HRP showed a substrate conversion of 72.56%, 80.81%, 65.75% and 52.57%, respectively. The HRP&Au@ZIF-8 showed a conversion of 86.61%, 90.96%, 73.16% and 69.53%, respectively. And the HRP&Au@ZIF-8/cDNA showed a substrate conversion of 84.38%, 90.89%, 73.32% and 66.65%, respectively. Obviously, HRP&Au@ZIF-8 and HRP&Au@ZIF-8/cDNA composites showed higher conversion rates than those of free HRP in acidic and alkaline conditions. It may be due to the fact that ZIF-8’s rigid structure suppressed the unfolding and aggregation of HRP, thereby maintaining the original structure of HRP molecules [38]. The aforementioned results revealed that HRP&Au@ZIF-8 and HRP&Au@ZIF-8/cDNA composites could tolerate extreme conditions and maintain high catalytic activity. These enable reliable detection of DON in harsh conditions, e.g., during cereal transportation or in remote resource-limited areas where the environmental factors may be uncontrollable.

To verify the feasibility of HRP&Au@ZIF-8/cDNA as a signal tag, DON with a concentration of 200 ng/mL was detected using the proposed ELAA. As shown in Figure S6, without the addition of DON, there was an obvious peak at 450 nm. It was primarily attributed to the complementary binding of HRP&Au@ZIF-8/cDNA to the biotin-modified Apt that was immobilized on the microplates. In the presence of DON, DON in samples was firstly captured by DON Apt, and the unbound Apt hybridized with the HRP&Au@ZIF-8/cDNA signal tags. Owing to the fact that abundant Apt binding sites were occupied by DON, the number of signal tags on the microplates decreased, resulting in a decreased peak absorbance. It demonstrated that the colorimetric ELAA based on HRP&Au@ZIF-8/cDNA as signal tags was feasible.

### 3.3. Optimization of Detection Parameters

**Figure 2:**
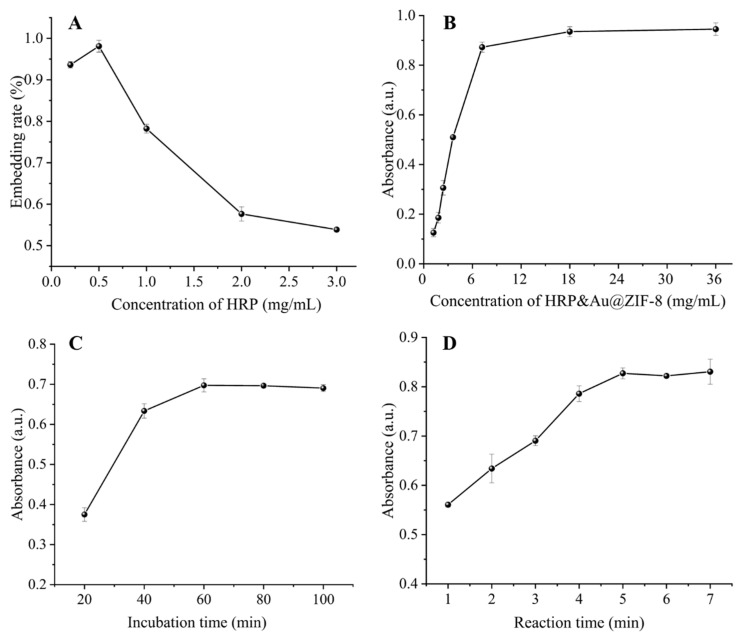
Optimization of experimental parameters: HRP embedding concentration in signal tag (**A**), HRP&Au@ZIF-8/cDNA concentration (**B**), incubation time of HRP&Au@ZIF-8/cDNA (**C**) and reaction time of TMB oxidation (**D**). The error bar represents the standard deviation of three repeated experiments.

For better analytical performance, several experimental parameters were optimized. The concentration of HRP encapsulated in the signal tag affected the sensitivity and detection limit for DON. To determine the optimal HRP concentration and its packaging efficiency, various concentrations of HRP were embedded with a fixed amount of ZIF-8. With the increase of HRP concentrations from 0.2 mg/mL to 0.5 mg/mL in Figure 2A, the entrapment rates increased from 92.6% to 98.1% due to the increased number of HRP-triggered ZIF-8 nucleation sites. When further increasing the concentration of HRP to 3 mg/mL, the entrapment rate decreased as a result of the saturation of nucleation sites. Therefore, the optimized HRP concentration was 0.5 mg/mL. Then, we optimized the concentration and incubation time of HRP&Au@ZIF-8/cDNA probes. The concentration of the HRP&Au@ZIF-8/cDNA probe is crucial for the signal intensity. As shown in Figure 2B, the absorbance initially increased with the increase of HRP&Au@ZIF-8/cDNA concentrations and thereafter reached a stable plateau when further raising the concentration to 7.2 mg/mL. Therefore, the HRP&Au@ZIF-8/cDNA concentration was optimized to 7.2 mg/mL. The incubation time between Apt and the HRP&Au@ZIF-8/cDNA probe is very important. Figure 2C shows that the absorbance at 450 nm increased gradually with the increase of incubation time of HRP&Au@ZIF-8/cDNA probes, and then reached the maximum value and stabilized at 60 min. Therefore, a 60-minute incubation time was required for a sufficient reaction between Apt and HRP&Au@ZIF-8/cDNA probes. Finally, the catalytic time of TMB oxidation was also optimized. It can be seen from Figure 2D that the absorbance increased with the increase of catalysis time and began to level off after 5 min. Therefore, a 5-minute reaction time was used in the subsequent experiment for the catalytical oxidation of TMB.

### 3.4. Performance of the Colorimetric ELAA Based on the HRP&Au@ZIF-8/cDNA Composites for DON Detection

**Figure 3:**
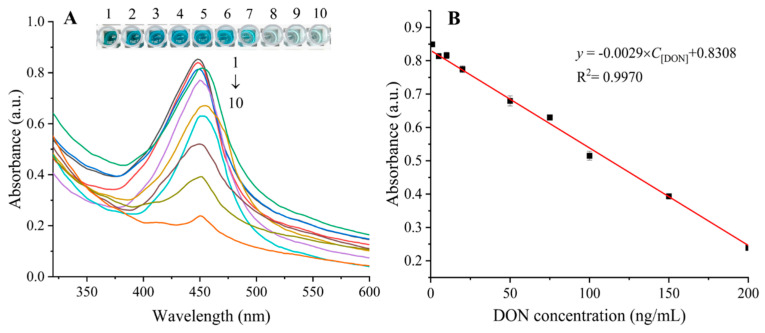
Absorption spectra in the presence of DON with different concentrations (**A**), DON concentrations from top to bottom (1–10) were 0, 1, 5, 10, 20, 50, 75, 100, 150 and 200 ng/mL, respectively. Linear plot of absorbance vs. DON concentration (**B**). Error bars indicated the standard deviation of three measurements.

Under the optimized conditions, different concentrations of DON were detected to assess the performance of the proposed biosensor. As shown in Figure 3A, the colors of the samples visibly changed from dark blue to light blue as the concentration of DON increased from 0 to 200 ng/mL. After stopping the reaction with the addition of H_2_SO_4_, the absorbance values at 450 nm decreased accordingly with the increase of DON concentrations in the sample. The relationship between the absorbance at 450 nm and DON concentration was shown in Figure 3B, in which a linear relationship was established over the dynamic DON concentration range of 1–200 ng/mL. The regression equation between the absorbance at 450 nm (*y*) and the DON concentration (*C*_[DON]_, ng/mL) could be described as *y* = − 0.0029*C*_[DON]_ + 0.8308 (*R*^2^ = 0.9970). The limit of detection (LOD), defined as 3*SD*/*b* (*SD* represents the standard deviation of the colorimetric signal response, and *b* represents the slope of the calibration curve), was calculated to be 0.5068 ng/mL. The analytical performance of some reported methods and the proposed ELAA were compared in Table S2. Overall, the proposed ELAA displayed a lower LOD in comparison with those of other methods, indicating that the developed ELAA had excellent analytical performance for DON. The proposed method exhibited several advantages over other analytical methods. First, the large surface area, open structure and high porosity of ZIF-8 improved the loading rates of cDNA and HRP, which helped to accelerate the reaction and amplify the detection signal and thus enhance the detection sensitivity. Second, the protective ZIF-8 coating on the enzyme surface improved the resistance to harsh environments and enhanced the stability of the methods.

### 3.5. Evaluation of the Assay Specificity

**Figure 4:**
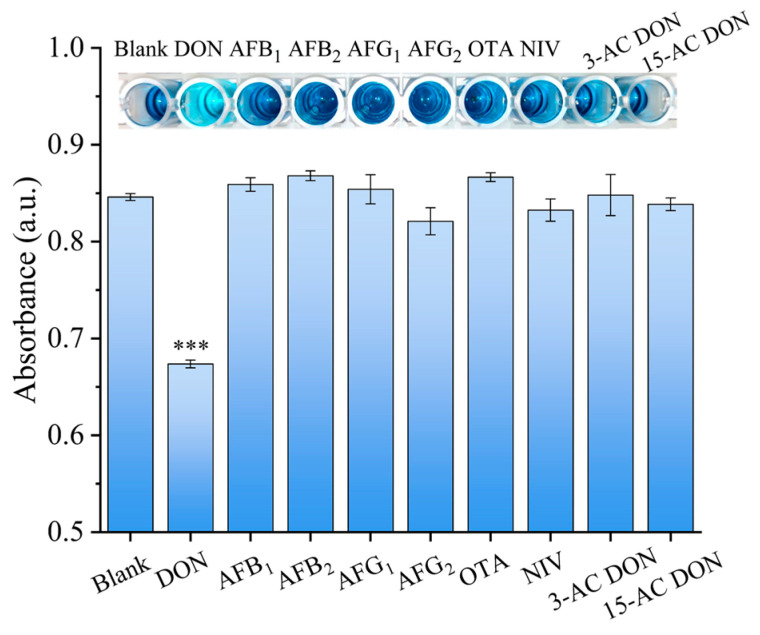
Selectivity of DON detection. *** indicates a statistically significant difference between the DON samples and the interfering samples (*p* ≤ 0.01). Error bars represent the standard deviation of the three experiments.

To investigate the selectivity of the established ELAA colorimetric method, AFB_1_, AFB_2_, AFG_1_, AFG_2_, OTA, NIV, 3-ACDON and 15-ACDON were used as interfering reagents. The concentration of the interferents was 500 ng/mL, and the concentration of DON was decreased to 50 ng/mL. The inset of Figure 4 shows that the interfering mycotoxins displayed a distinct dark blue color, which was similar to that of the blank control. By contrast, the color of the solution containing DON was significantly lighter than those of other mycotoxins, indicating that the samples containing DON could be easily differentiated by visual inspection. Statistical analysis showed that the absorbance values between DON and the other mycotoxins were significantly different (*p* < 0.05), revealing the high specificity of the developed ELAA for DON.

### 3.6. Stability Analysis

The stability of the colorimetric ELAA method was explored. To assess its thermostability, HRP&Au@ZIF-8/cDNA composites were heat-treated at 40 °C for various durations and then used as signal tags for the detection of DON (10 ng/mL). As shown in Figure S7A, no obvious difference in the detection signals was observed, showing that the proposed ELAA method had good heat resistance. Then, the HRP&Au@ZIF-8/cDNA signal tags were stored at a 4 °C refrigerator for a period of time and used for DON detection every three days to evaluate their storage stability. As displayed in Figure S7B, during the storage, the RSDs of the detection signals for 10 ng/mL DON were still within 4%, demonstrating the long-term stability of the method.

### 3.7. Actual Cereal Sample Analysis

In order to assess the feasibility of the proposed colorimetric ELAA method for actual sample analysis, rice, wheat and maize flour samples were spiked with different concentrations of DON and detected by the developed method. The DON concentration in the spiked cereal samples was calculated based on the aforementioned fitted regression equation, and the results are shown in Table 1. The recovery rates of rice flour samples containing 1 ng/mL, 10 ng/mL, 50 ng/mL, 100 ng/mL and 200 ng/mL DON ranged from 98% to 104% with a relative standard deviation (RSD) range of 1.33%–4.95% (*n* = 3). The recovery rates of wheat flour samples ranged from 96% to 110% with an RSD range of 1.06%–4.55% (*n* = 3). As for the maize flour samples, the recovery rates ranged from 86% to 105% with an RSD range of 0.72%–3.92% (*n* = 3). The matrix effects of rice, wheat and maize flour were almost negligible, and the RSDs of the spiked recoveries were all less than 5%. In order to further evaluate the practicability of the proposed ELAA for DON detection in food samples, a commercialized ELISA kit was also used to analyze DON-spiked samples for comparison. Table 1 shows that the results obtained from the developed ELAA method showed good agreement with those obtained from the ELISA kit, implying that the proposed ELAA owned high reliability. Moreover, DON-positive wheat and maize flour samples, which had been confirmed by HPLC, were also detected by the developed ELAA. As shown in Table S3, the detection results obtained from ELAA displayed high consistency with the HPLC results, further revealing the practicability of this method. Compared with the traditional antibodies, it was worth noting that the use of aptamers in this study avoided the complicated preparation of bioreceptors and decreased the detection cost, while they did not compromise the detection sensitivity. In contrast with the commercial ELISA kit (Table S4), this method was cost-effective (< USD1 per test) and displayed a lower LOD. With a wide linear detection range and low LOD, the developed ELAA held great significance for massive detection in practical applications.

## 4. Conclusions

In summary, a novel ELAA was developed to detect DON using HRP&Au@ZIF-8/cDNA as a signal tag. The rigid structure of ZIF-8 promoted the tolerance of the encapsulated HRP to harsh environments, significantly improving the stability of the HRP enzyme. Based on the developed ELAA, satisfactory analytical performance including high sensitivity, good specificity and excellent stability was obtained for DON detection. Compared to other colorimetric methods, the proposed method exhibited a lower LOD of 0.5068 ng/mL. Moreover, the developed sensor could also be successfully applied for DON determination in the cereal samples and showed acceptable agreement with the results obtained from a commercial ELISA kit. The above results highlight the potential application of the ELAA method in the field of food safety.

## Figures and Tables

**Table 1:** Recovery rates of DON in the spiked cereal samples by the proposed ELAA and ELISA.

Samples	Spiked Concentration (ng/mL)	Measured Concentration (Mean ± SD ^a^) (ng/mL)	Recovery ^b^ (%)	RSD ^c^ (%)	ELISA Validation (Mean ± SD) (ng/mL)	Recovery (%)	RSD (%)
Rice flour	1	1.01 ± 0.05	101.15	4.95	−	−	−
	10	9.76 ± 0.13	97.59	1.33	10.01 ± 0.17	100.12	1.70
	50	52.00 ± 0.87	104.00	1.67	50.16 ± 0.79	100.31	1.57
	100	100.99 ± 2.20	100.99	2.18	109.85 ± 0.66	109.85	0.60
	200	198.59 ± 7.13	99.29	3.59	199.89 ± 1.33	99.95	0.67
Wheat flour	1	1.10 ± 0.05	110.34	4.55	−	−	−
	10	10.38 ± 0.11	103.77	1.06	10.10 ± 0.19	100.95	1.88
	50	50.91 ± 0.93	101.82	1.83	53.25 ± 0.69	106.49	1.30
	100	99.12 ± 1.41	99.12	1.42	100.84 ± 1.42	100.84	1.41
	200	191.99 ± 7.13	95.99	3.71	196.35 ± 3.36	98.18	1.71
Maize flour	1	0.86 ± 0.02	86.21	2.33	−	−	−
	10	9.75 ± 0.07	97.50	0.72	10.91 ± 0.19	109.07	1.74
	50	48.09 ± 1.04	96.18	2.16	51.56 ± 0.43	103.12	0.83
	100	101.88 ± 0.95	101.88	0.93	110.54 ± 2.80	110.54	2.53
	200	209.43 ± 8.21	104.71	3.92	201.75 ± 1.51	100.87	0.75

^a^ SD: standard deviation; ^b^ recovery rates (%) = (measured concentration/spiked concentration) × 100%; and ^c^ RSD (%) = standard deviation/mean value × 100%; −: outside the detection range.

## Data Availability

Data are contained within the article or Supplementary Materials.

## Supplementary Materials

The following supporting information can be downloaded at: https://www.mdpi.com/article/10.3390/bios13090847/s1, Figure S1: SEM image of AuNPs; Figure S2: EDS mapping, spectrogram and elemental content analysis of HRP&Au@ZIF-8; Figure S3: UV-vis absorption spectra of cDNA, ZIF-8, HRP&Au@ZIF-8 and HRP&Au@ZIF-8/cDNA; Figure S4: TGA curves of HRP, ZIF-8, HRP@ZIF-8 and HRP&Au@ZIF-8; Figure S5: The steady-state kinetic study of the HRP&Au@ZIF-8/cDNA composites; Figure S6: Colorimetric responses of the ELAA in the presence of 200 ng/mL DON; and Figure S7: Heat resistance (A) and storage stability (B) of the ELAA colorimetric method. Table S1: Sequence information of the oligonucleotides used in this work; Table S2: Comparison of different methods for DON detection; Table S3: Detection of DON in actual samples by the proposed ELAA method; and Table S4: Comparison of the detection limit and cost between ELISA and the proposed ELAA method. References [39,40,41,42,43,44,45] are cited in Supplementary Materials.
