# Supplementary material for: Zeolitic Imidazolate Framework-8 Composite-Based Enzyme-Linked Aptamer Assay for the Sensitive Detection of Deoxynivalenol
Source: Biosensors (Basel). 2023 Aug 25;13(9):847. doi: 10.3390/bios13090847 (PMC10526248; doi:10.3390/bios13090847)
Supplement: Supplementary file 1 [file biosensors-13-00847-s001.zip › biosensors-2549770-supplementary.pdf]

biosensors

MDPI

Supporting Information For

Zeolitic Imidazolate Framework-8 Composite-Based Enzyme-Linked Aptamer Assay for the Sensitive Detection of Deoxynivalenol

Zaixi Shu [1,2,†], Run Zhou [1,2,†], Guijie Hao [3], Xingyue Tang [1,3], Xin Liu [1,2], Jie Bi [1,2], Huang Dai [1,2,*] and Yafang Shen [3,*]

[1] College of Food Science and Engineering, Wuhan Polytechnic University, Wuhan 430023, China; shuzaixi@163.com (Z.S.); zhourun285@163.com (R.Z.); 15327248225@163.com (X.T.); liuxinhook@126.com (X.L.); bj15926225191@163.com (J.B.)

[2] Key Laboratory for Deep Processing of Major Grain and Oil, Wuhan Polytechnic University, Ministry of Education, Wuhan 430023, China

[3] Key Laboratory of Healthy Freshwater Aquaculture, Ministry of Agriculture and Rural Affairs, Key Laboratory of Fish Health and Nutrition of Zhejiang Province, Huzhou Key Laboratory of Aquatic Product Quality Improvement and Processing Technology, Zhejiang Institute of Freshwater Fisheries, Huzhou 313001, China; haoguijie2022@hotmail.com

* Correspondence: huangdai9@126.com (H.D.); shenyafang1994@126.com (Y.S.)

† These authors contributed equally to this work.

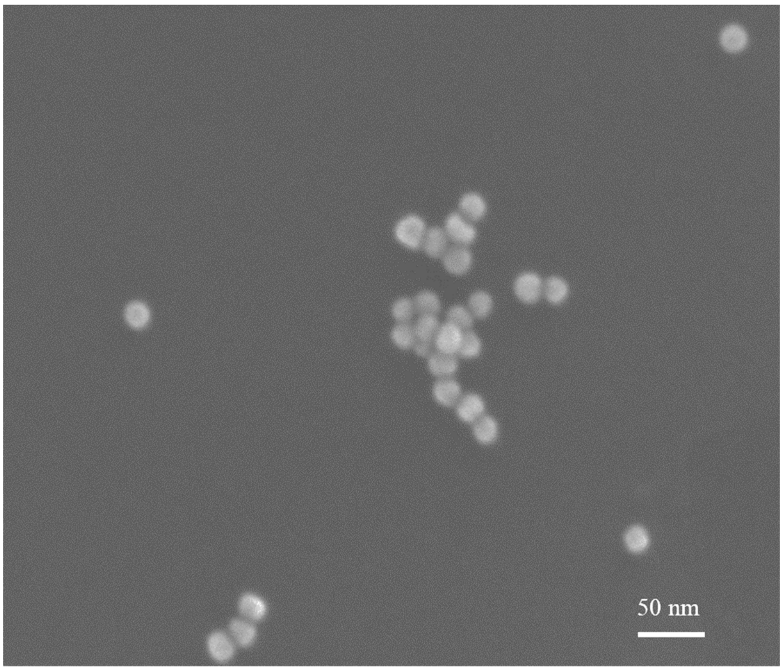

Figure S1. SEM image of AuNPs.

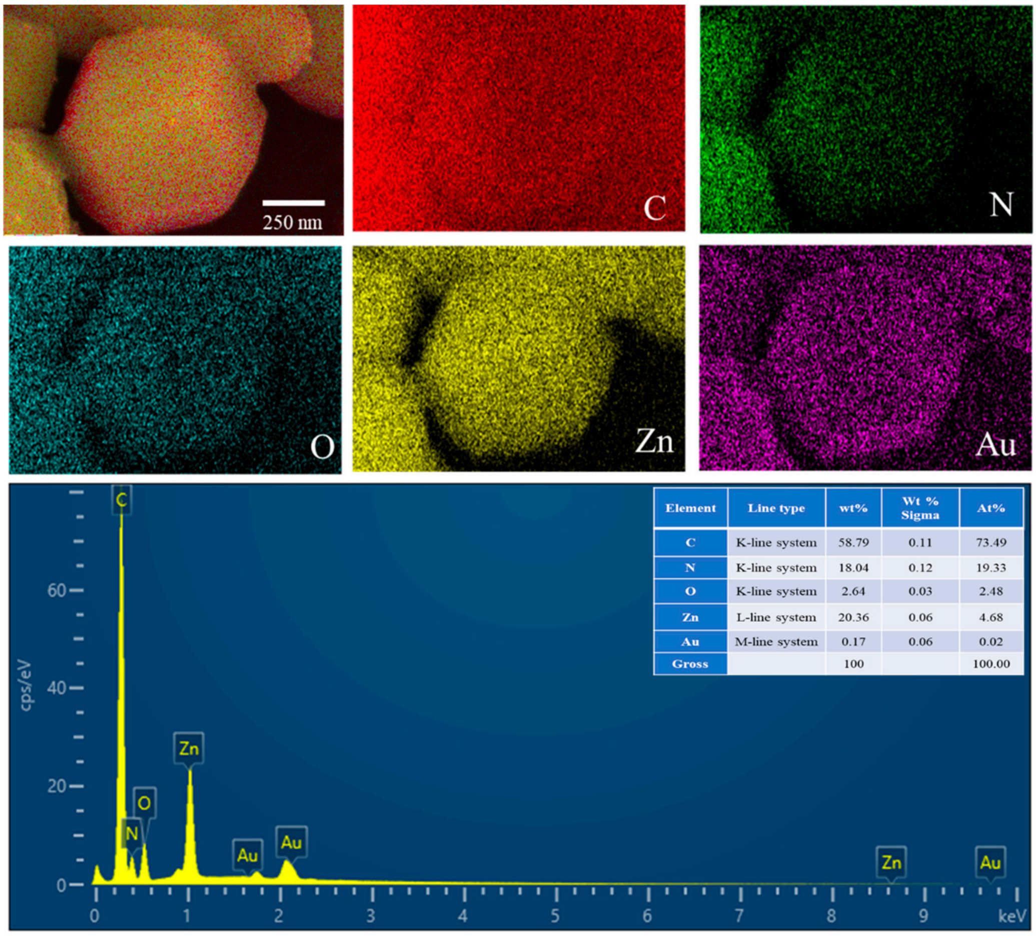

Element	Line type	wt%	Wt % Sigma	At%
C	K-line system	58.79	0.11	73.49
N	K-line system	18.04	0.12	19.33
O	K-line system	2.64	0.03	2.48
Zn	L-line system	20.36	0.06	4.68
Au	M-line system	0.17	0.06	0.02
Gross		100		100.00

Figure S2. EDS mapping, spectrogram and elemental content analysis of HRP&Au@ZIF-8.

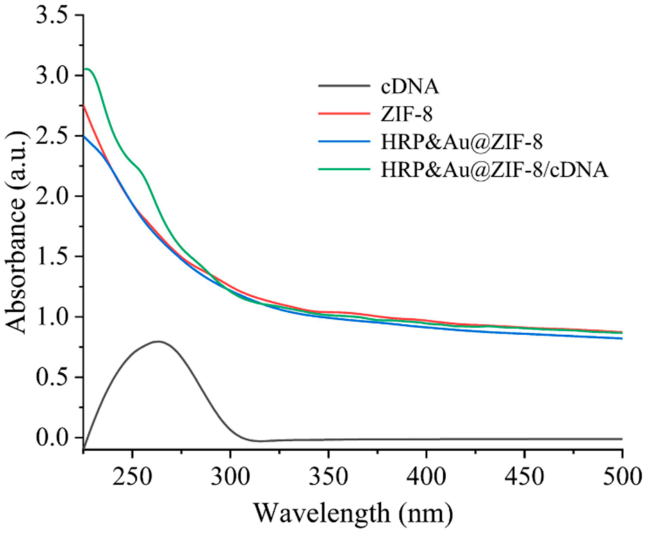

Figure S3. UV-vis absorption spectra of cDNA, ZIF-8, HRP&Au@ZIF-8 and HRP&Au@ZIF-8/cDNA.

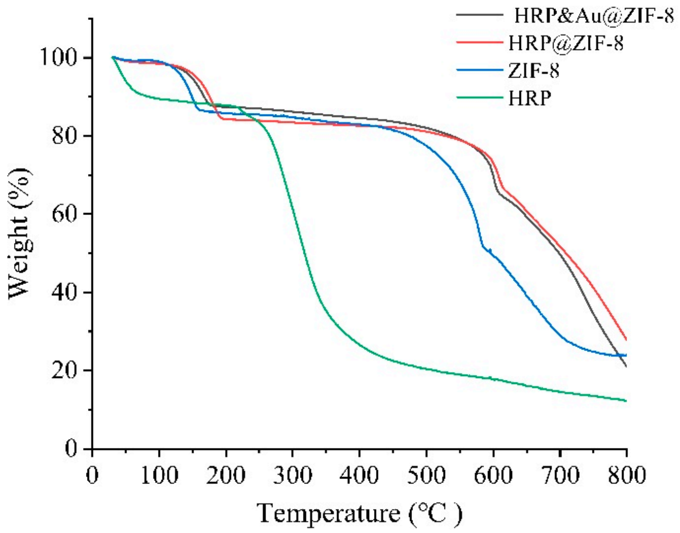

Figure S4. TGA curves of HRP, ZIF-8, HRP@ZIF-8 and HRP&Au@ZIF-8.

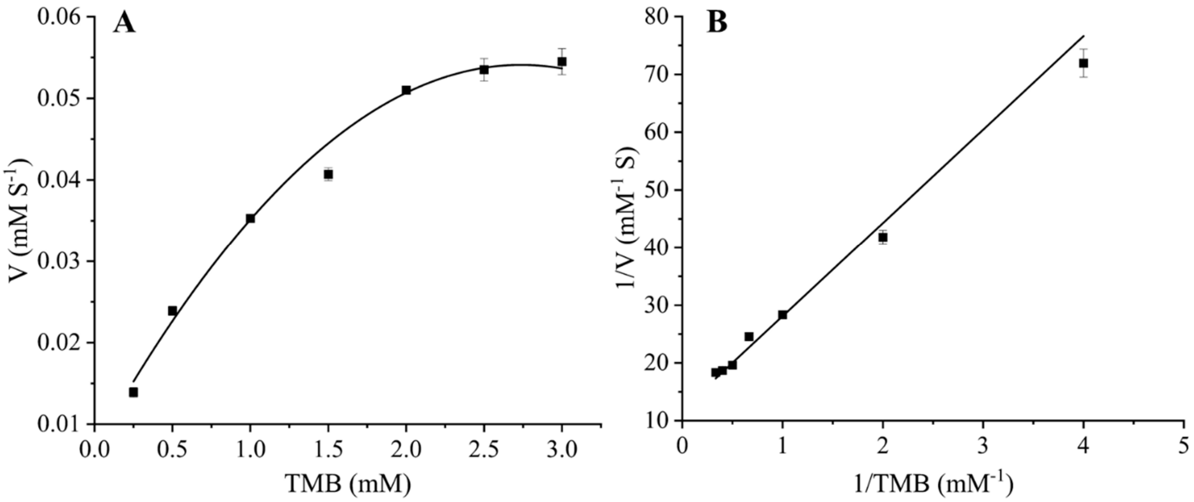

Figure S5. The steady-state kinetic study of the HRP&Au@ZIF-8/cDNA composites (0.024 mg/mL the composites, 2.642 mM H_2O_2). Plot of initial reaction rate versus TMB concentration (A) and the corresponding Lineweaver–Burk plots of the Michaelis–Menten equation (B).

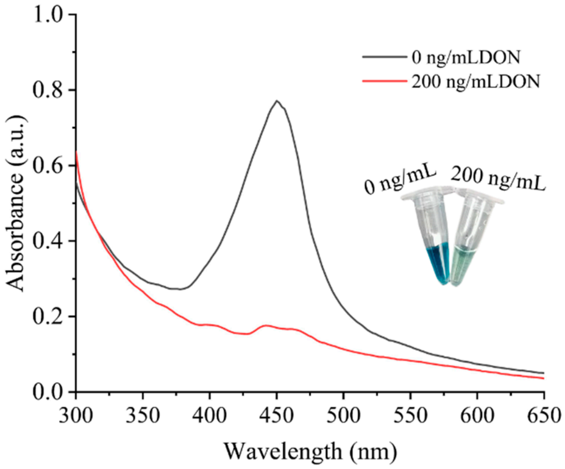

Figure S6. Colorimetric responses of the ELAA in the presence of 200 ng/mL DON.

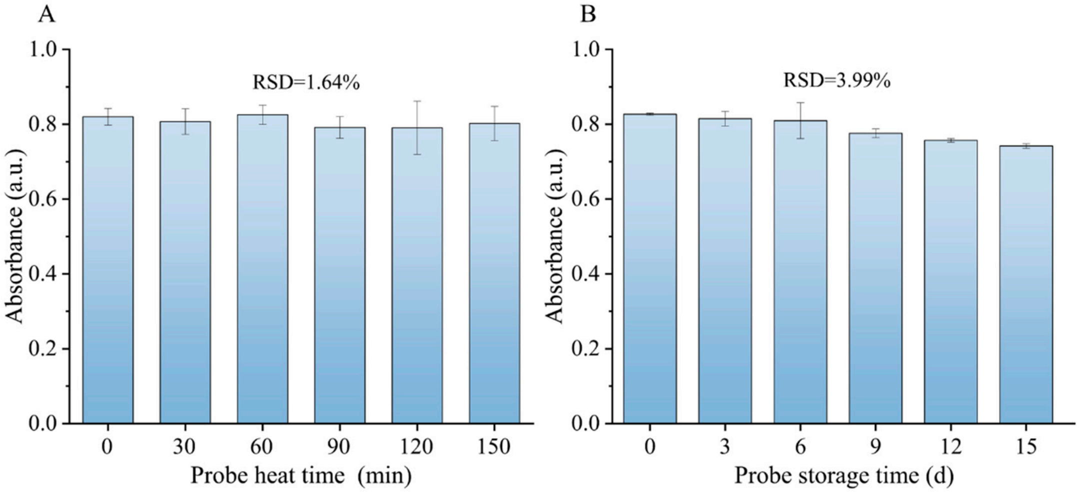

Figure S7. Heat resistance (A) and storage stability (B) of the ELAA colorimetric method.

Table S1. Sequence information of the oligonucleotides used in this work.

Name	Sequence (from 5' to 3')
DON Aptamer	Biotin-GCA TCA CTA CAG TCA TTA CGC ATC GTA GGG GGG ATC GTT AAG GAA GTG CCC GGA GGC GGT ATC GTG TGA AGT GCT GTC CC
cDNA	SH C6-GGG ACA GCA CTT CAC ACG ATA CCG CCT CCG GGC ACT TCC TTA ACG ATC CCC CCT ACG ATG CGT AAT GAC TGT AGT GAT GC
single-stranded DNA	AAAAAAAA

Table S2. Comparison of different methods for DON detection.

Methods	Materials	Sample matrices	Linear range (ng/mL)	LOD (ng/mL)	Reference
Colorimetric competitive immunoassay	DON-Chip	Corn	10–20000	4.7	[1]
Lateral flow assay	Antibody-nano-Au	Corn	–	10	[2]
Multiplex immunosorbent assay	QD@SiO_2	Wheat and maize	55–420	35	[3]
Magnetic immunoassay	Au NBPs	Wheat and maize	0–2000	57.93	[4]
DPV	Bi_2O_3 modified SPCEs	Rice	510–1400	7.1	[5]
TRFIA	Europium ion polystyrene fluorescence microspheres	Wheat and corn	–	170	[6]
LFA	QB-DON mAbs	Feed	–	0.5	[7]
Enzyme-linked aptamer assay	HRP&Au@ZIF-8	Rice, wheat and maize	1–200	0.5	This work

QD: quantum dots; DPV: differential pulse voltammetry; TRFIA: Time-resolved fluorescence microsphere immunoassay; SPCEs: screen-printed carbon electrodes; FLA: Fluorometric lateral flow immunoassay; QBs: Quantum dot microbeads; ZIF-8: zeolitic imidazolate framework-8; mAbs: monoclonal antibodies

Table S3. Detection of DON in actual samples by the proposed ELAA method (*n*=3).

Sample	DON concentration found by HPLC (ng/mL)	Measured concentration (mean ± SD) (ng/mL)
Wheat flour	28	29.31±0.69
Maize flour	188	193.79±2.57

HPLC: high performance liquid chromatography.

Table S4. Comparison of the detection limit and cost between ELISA and the proposed ELAA method.

Method	LOD (ng/mL)	Cost per test ($)
commercial ELISA kit	5.6313	1.83
the proposed ELAA method	0.5068	<1

The cost of ELAA was estimated by the reagent and consumables.

References

[1] Q. Jiang, J.D. Wu, K. Yao, Y.L. Yin, M.M. Gong, C.B. Yang, F. Lin, Paper-based microfluidic device (DON-Chip) for rapid and low-cost deoxynivalenol quantification in food, feed, and feed ingredients, ACS Sensors 4 (2019) 3072-3079, https://doi.org/10.1021/acssensors.9b01895.

[2] S.C. Yu, L.L. He, F. Yu, L. Liu, C.L. Qu, L.B. Qu, J. Liu, Y.M. Wu, Y.J. Wu, A lateral flow assay for simultaneous detection of Deoxynivalenol, Fumonisin B_1 and Aflatoxin B_1, Toxicon 156 (2018) 23-27, https://doi.org/10.1016/j.toxicon.2018.10.305.

[3] N. Beloglazova, P. Lenain, M. Tessier, I. Goryacheva, Z. Hens, S. De Saeger, Bioimprinting for multiplex luminescent detection of deoxynivalenol and zearalenone, Talanta 192 (2019) 169-174, https://doi.org/10.1016/j.talanta.2018.09.042.

[4] R. Guo, Y. Ji, J.N. Chen, J. Ye, B.X. Ni, L. Li, Y. Yang, Multicolor visual detection of deoxynivalenol in grain based on magnetic immunoassay and enzymatic etching of plasmonic gold nanobipyramids, Toxins 15 (2023) 351, https://doi.org/10.3390/toxins15060351.

[5] R. Maria-Hormigos, M.J. Gismera, M.T. Sevilla, A. Rumbero, J.R. Procopio, Rapid and easy detection of deoxynivalenol on a bismuth oxide screen-printed electrode, Electroanalysis 29 (2017) 60-66, https://doi.org/10.1002/elan.201600484.

[6] J.D. Sun, L.Z. Wang, J.D. Shao, D.D. Yang, X.R. Fu, X.L. Sun, One-step time-resolved fluorescence microsphere immunochromatographic test strip for quantitative and simultaneous detection of DON and ZEN, Analytical and Bioanalytical Chemistry 413 (2021) 6489-6502, https://doi.org/10.1007/s00216-021-03612-0.

[7] R.X. Li, C.Z. Meng, Y. Wen, W. Fu, P.L. He, Fluorometric lateral flow immunoassay for simultaneous determination of three mycotoxins (aflatoxin B_1, zearalenone and deoxynivalenol) using quantum dot microbeads, Microchimica Acta 186 (2019) 748, https://doi.org/10.1007/s00604-019-3879-6.
